# Identification of Novel Antistaphylococcal Hit Compounds Targeting Sortase A

**DOI:** 10.3390/molecules26237095

**Published:** 2021-11-24

**Authors:** Galyna Volynets, Hanna Vyshniakova, Georgiana Nitulescu, George Mihai Nitulescu, Anca Ungurianu, Denisa Margina, Olena Moshynets, Volodymyr Bdzhola, Ihor Koleiev, Olga Iungin, Sergiy Tarnavskiy, Sergiy Yarmoluk

**Affiliations:** 1 Institute of Molecular Biology and Genetics, The NAS of Ukraine, 150 Zabolotnogo St., 03143 Kyiv, Ukraine; g.p.volynets@gmail.com (G.V.); moshynets@gmail.com (O.M.); Volodymyr_Bdzhola@ukr.net (V.B.); olgaungin@gmail.com (O.I.); starn999@gmail.com (S.T.); sergiy@yarmoluk.org.ua (S.Y.); 2 Scientific Services Company Otava Ltd., 150 Zabolotnogo St., 03143 Kyiv, Ukraine; 3 L.V. Gromashevsky Institute of Epidemiology and Infectious Diseases NAMS of Ukraine, 5 Amosova St., 03038 Kyiv, Ukraine; hv.vyshniakova@gmail.com; 4 Faculty of Pharmacy, “Carol Davila” University of Medicine and Pharmacy, Traian Vuia 6, 020956 Bucharest, Romania; george.nitulescu@umfcd.ro (G.M.N.); anca.ungurianu@umfcd.ro (A.U.); denisa.margina@umfcd.ro (D.M.); 5 Educational and Scientific Center “Institute of Biology and Medicine”, Taras Shevchenko National University of Kyiv 64/13, Volodymyrska Str., 01601 Kyiv, Ukraine; koleev.igor@gmail.com

**Keywords:** *Staphylococcus aureus*, sortase A, molecular docking, inhibitor, antibiotic resistance

## Abstract

*Staphylococcus aureus* (*S. aureus*) is a causative agent of many hospital- and community-acquired infections with the tendency to develop resistance to all known antibiotics. Therefore, the development of novel antistaphylococcal agents is of urgent need. Sortase A is considered a promising molecular target for the development of antistaphylococcal agents. The main aim of this study was to identify novel sortase A inhibitors. In order to find novel antistaphylococcal agents, we performed phenotypic screening of a library containing 15512 compounds against *S. aureus* ATCC43300. The molecular docking of hits was performed using the DOCK program and 10 compounds were selected for in vitro enzymatic activity inhibition assay. Two inhibitors were identified, N,N-diethyl-N′-(5-nitro-2-(quinazolin-2-yl)phenyl)propane-1,3-diamine (**1**) and acridin-9-yl-(1*H*-benzoimidazol-5-yl)-amine (**2**), which decrease sortase A activity with IC_50_ values of 160.3 µM and 207.01 µM, respectively. It was found that compounds **1** and **2** possess antibacterial activity toward 29 tested multidrug resistant *S. aureus* strains with MIC values ranging from 78.12 to 312.5 mg/L. These compounds can be used for further structural optimization and biological research.

## 1. Introduction

*Staphylococcus aureus* (*S. aureus*) is a causative agent of the majority of skin infections, hospital-acquired infections and severe diseases such as bacteremia, sepsis, meningitis, osteomyelitis, endocarditis, and pneumonia [1].

*S. aureus* belongs to the group of high-priority dangerous “ESKAPE” pathogens, which includes the multidrug resistant isolates of *Enterococcus*, *Staphylococcus*, *Klebsiella*, *Acinetobacter*, *Pseudomonas* and *Enterobacter* that are currently considered as the greatest challenge in medicine. Many hospital-acquired *S. aureus* isolates have become resistant to most available antibiotics. Staphylococcal resistance to penicillin is mediated by penicillinase which hydrolyses the β-lactam ring of antibiotic. In clinical practice for treatment of staphylococcal infections, methicillin, chemically modified penicillin which cannot be hydrolyzed by penicillinase, has been widely used. At the present time, methicillin-resistant *S. aureus* (MRSA), which is resistant to all of the β-lactam antibiotics due to modification of penicillin-binding protein, has become one of the most dangerous pathogens. The antibiotic of choice in a case of MRSA-associated infection is vancomycin. However, vancomycin-resistant MRSA strains have emerged recently [2]. It should be noted that no other antibiotic to date has shown any superiority to vancomycin in the treatment of MRSA infections with the possible exception of linezolid in hospital-acquired pneumonia [3]. Therefore, the development of multidrug resistance in *S. aureus* has caused an urgent need for the search of novel effective targets and corresponding inhibitors to develop principally new antibiotics effective against strains which are resistant to already known antibiotics of choice.

Sortase A is considered as a promising molecular target for the development of antistaphylococcal agents. Sortase A is a membrane-bound transpeptidase which catalyzes the transfer and immobilization of essential virulence factors to the surface of microorganisms. Inhibitors of sortase A affect virulence and biofilm formation, therefore decreasing selective pressure which can cause the development of antibiotic resistance [4]. Sortase A is not presented in eukaryotic organisms, hence the inhibitors of this enzyme may possibly have less toxicity for human organisms. Since this enzyme is membrane-bound, the inhibitors do not have to penetrate into the cell. 

To date, small-molecular inhibitors of sortase A have been reported among the derivatives of diarylacrylonitrile [5,6], aryl(β-amino)ethyl ketone [7], rhodanine, pyridazinone, pyrazolethione [8], morpholinobenzoate, aryl 3-acryloamides [9,10], dihydro-β-carboline [11], benzo[d]isothiazol-3(2*H*)-one-adamantane amine [12], 3,6-disubstituted triazolothiazole [13], 2-phenylbenzofuran-3-carboxamide [14], 2-phenylbenzo[d]oxazole-7-carboxamide [15], 2-(2-phenylhydrazinylidene)alkanoic acid [16], indolethiazolidine [17], pyrrolomycin [18], 2-phenylthiazole [19], 2,5-disubstituted thiadiazole [20], thiadiazolinedione [21], disulfanylbenzamide [22], and 1,2,4-oxadiazole topsentin analogs [23]. Furthermore, several inhibitors for sortase A have been identified among natural products such as β-sitosterol-3-O-glucopyranoside [24], berberine chloride [25], bis(indole)-alkaloid [26], isoaaptamine [27], flavonoids (kurarinol [28], myricetin [29], quercitrin [30], morin [31], eriodictyol [32], acacetin [33], 7-hydroxy-6-methoxy-flavanone and formononetin [34], dryocrassin ABBA [35]), curcumin [36], maltol-3-O-(4′-O-cis-p-cumaroyl-6′-O-(3-hydroxy-3-methylglutaroyl)-*β*-glucopyranoside [37], skyrin [38], aspermytin A [39], natural naphthoquinones [40], isovitexin [41], coumarines [42], taxifolin [43], erianin [44], quinone [45], chalcone [46], bis(indole) alkaloids [47], orientin [48], gallotannins [49] and peptides from the marine-derived fungi *Aspergillus allahabadii* [50]. Several review works describe important small organic compounds that act as potent sortase A inhibitors [51,52,53,54]. In the present article, we report two novel inhibitors of *S. aureus* sortase A belonging to novel chemical classes, the derivatives of acridin-9-yl-(1*H*-benzoimidazol-5-yl)-amine and 2-phenyl-quinazoline, which possess antimicrobial activity toward multidrug resistant *S. aureus* strains.

## 2. Results

In order to find novel antistaphylococcal agents, phenotypic screening of a library containing 15,512 compounds, provided by commercial supplier OTAVA Ltd., was performed by the Community for Open Antimicrobial Drug Discovery (CO-ADD), against methicillin-resistant *S aureus* strain ATCC43300. As a result, 250 compounds inhibiting growth of MRSA ATCC43300 at least by 30% at the concentration of 32 mg/L were found. The list of active compounds in SMILES format is available in the Supplementary Materials. To identify which compounds can potentially inhibit *S. aureus* sortase A, we performed molecular docking of 250 compounds into the active site of this enzyme using the DOCK program. According to the results of molecular docking calculations and visual inspection of the best-scored complexes, we selected 10 compounds for in vitro testing. The chemical structure of compounds and their antibacterial activity toward *Staphylococcus aureus* are presented in Table 1. 

The tested compounds inhibited the growth of the methicillin-resistant *S. aureus* strain ATCC43300 with inhibition percentages ranging from 38.98 up to 99.57%. Compounds **2** and **4** almost completely inhibited the bacterial growth after exposure at 32 mg/L and could be used to develop new antibacterial substances.

### 2.1. Sortase A Activity Assay

**Figure 1:**
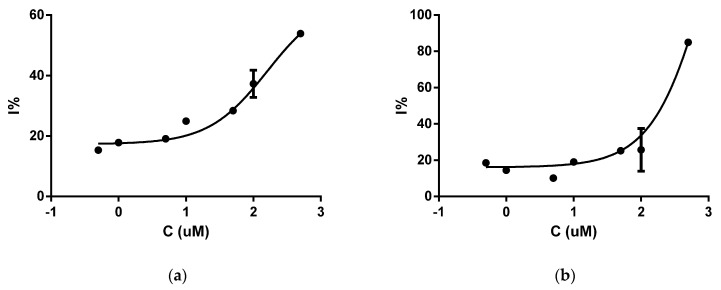
The plotted sortase A inhibition (I%) against compounds logarithm of concentration (μM): (**a**) compound **1**; (**b**) compound **2**.

Among these ten investigated compounds, using in vitro sortase A activity assay we found two inhibitors of *S. aureus* sortase A, N,N-diethyl-N′-(5-nitro-2-(quinazolin-2-yl)phenyl)propane-1,3-diamine (**1**) and acridin-9-yl-(1*H*-benzoimidazol-5-yl)-amine (**2**), which decreased enzyme activity with IC_50_ values of 160.3 µM and 207.01 µM, respectively (Figure 1). The other eight compounds produced only a small inhibition. Therefore, these compounds possess other molecular mechanisms of action. Possibly, compound **4**, with high antibacterial activity, should be investigated for inhibitory activity toward *S. aureus* DNA gyrase, since according to recent literature data, several indole derivatives demonstrate antistaphylococcal activity targeting this enzyme [55].

### 2.2. Molecular Docking

**Figure 2:**
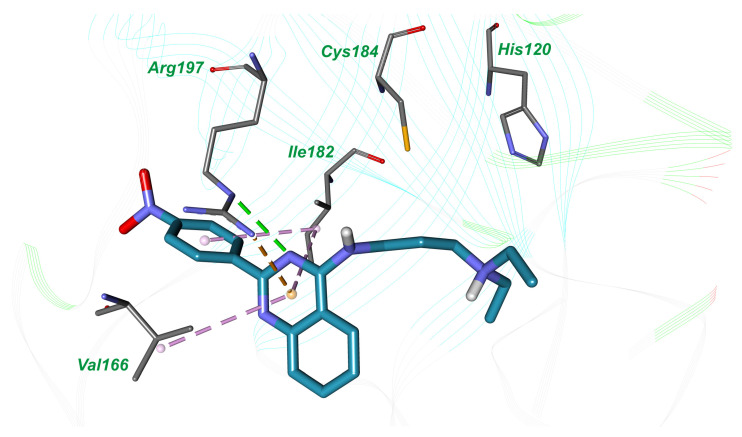
The binding mode of compound **1** in the active site of *S. aureus* sortase A; hydrogen bond is shown by the green dotted line, hydrophobic interactions are indicated by the magenta dotted lines and π-cation interaction is presented by orange dotted line.

According to molecular docking results, the compound N,N-diethyl-N′-(5-nitro-2-(quinazolin-2-yl)phenyl)propane-1,3-diamine (**1**) is involved in hydrophobic interactions with amino acid residues Val166 and Ile182 in the active site of sortase A. Furthermore, the nitrogen atom of the quinazolin ring forms a hydrogen bond with conserved amino acid residue Arg197, which is important for catalysis and belongs to catalytic triad (His120, Cys184, and Arg197). Quinazolin heterocycle is also implicated in π-cation interaction with Arg197 (Figure 2). 

**Figure 3:**
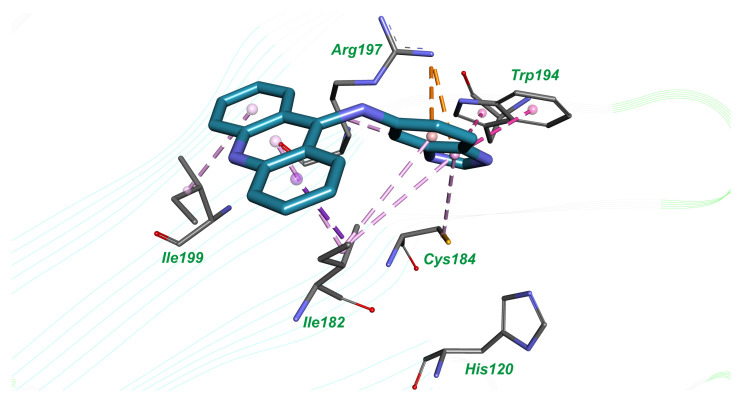
The binding mode of compound **2** in the active site of *S. aureus* sortase A; hydrogen bond is shown by the green dotted line, hydrophobic interactions are indicated by the magenta dotted lines and π-cation interactions are presented by the orange dotted lines.

The inhibitor acridin-9-yl-(1*H*-benzoimidazol-5-yl)-amine is involved in hydrophobic interactions with amino acid residues Ile199, Ile182, Cys184, Trp194 and benzoimidazol heterocycle forms π-cation interactions with Arg197 (Figure 3). 

### 2.3. Antibacterial Assay

The compounds **1** and **2** were extensively screened for antibacterial activity toward multidrug resistant *S. aureus* strains isolated in Ukrainian hospitals. The minimum inhibitory concentration (MIC) values for compounds **1** and **2** toward *S. aureus* isolates are presented in the Table 2.

The MIC values ranged between 78.12 and 312.5 mg/L for compound **1** and in the range of 156.2 and 312.5 mg/L for compound **2**. Compound **1** was more active than compound 2 on 12 of the 29 bacterial strains, while compound 2 had a higher antimicrobial effect on 3 strains. This observation is correlated with the higher inhibitory effect of compound **1** on the bacterial sortase.

The bacterial strains were investigated for sensitivity to antibiotics by the disco diffusion method using Mueller–Hinton Agar. The visual analysis of antibiotic sensitivity was performed according to EUCAST (European Committee on Antimicrobial Susceptibility Testing) recommendations [56] and the results are presented in Table 3.

## 3. Discussion

Using molecular docking techniques, we identified two novel inhibitors of *S. aureus* sortase A, N,N-diethyl-N′-(5-nitro-2-(quinazolin-2-yl)phenyl)propane-1,3-diamine (**1**), and acridin-9-yl-(1*H*-benzoimidazol-5-yl)-amine (**2**), which possess moderate enzyme inhibitory activity with IC_50_ values of 160.3 µM and 207.01 µM, respectively. When comparing the binding mode of highly potent benzisothiazolinone-based inhibitor in the crystal structure of *S. aureus* sortase A (PDB ID: 2MLM) [12], which was used for molecular docking, and docked complexes of compounds **1** (Figure 1) and **2** (Figure 2), it can be concluded that the simultaneous formation of a hydrogen bond with conservative Arg197 and tight hydrophobic interactions with Trp194, which were established in the co-crystal structure, can be important for inhibitory potency. It should be noted that in our study, compounds **1** and **2** form only one from these two intermolecular interactions—compound **1** builds hydrogen bond with Arg197 and compound **2** forms hydrophobic interactions with Trp194. Possibly, further chemical optimization of compounds **1** and **2** should be performed in order to reach both these types of ligand–receptor interactions. 

Antibacterial studies revealed that compounds **1** and **2** inhibit growth of a number of tested multidrug resistant *S. aureus* strains with MIC values in the range from 78.12 to 312.5 mg/L (Table 2). The antibacterial activity of sortase A inhibitors is low in comparison to known antistaphylococcal antibiotics such as vancomycin and linezolid, which have MIC values about 1 mg/L. Taking into account high levels of resistance to standard antibiotics, the development of antibiotics with novel mechanisms of action is of urgent need.

As it can be seen from the Table 2, compound **1** is more profitable than compound **2** and reveals the antimicrobial activity toward *S. aureus* strains which have different profiles of antibiotic sensitivity with MIC values in the range from 78.12 to 312.5 mg/L, while compound **2** demonstrates antimicrobial activity with MIC values in the range from 156.2 to 312.5 mg/L (Table 3).

Compound **1** possess the highest antibacterial activity with MIC value of 78.12 mg/L toward multidrug resistant *S. aureus* strains 1012, 501, 502, 504, 506, 510, 511. All these strains have susceptibility to chloramphenicol, moxifloxacin and linezolid. Vice versa, it was revealed that the compound **1** has the lowest antibacterial activity toward *S. aureus* isolates 964, 997 and 1013 which all have resistance to chloramphenicol; two of them (964, 997) have resistance to moxifloxacin and one of them (997) has resistance to linezolid. Compound **2** has antibacterial activity toward tested *S. aureus* strains, mostly with a MIC value of 156.2 mg/L, except three isolates, 854, 890 and 892, which all are susceptible to linezolid. Therefore, compounds **1** and **2** possess different effectiveness toward *S. aureus* strains with various antibiotic resistance profiles and can be useful for further optimization and development of novel lead compounds with antibacterial activity toward multidrug resistant *S. aureus* strains.

## 4. Materials and Methods

### 4.1. Sortase A Activity Assay

The inhibitory activity of compounds was determined by quantifying the fluorescence intensity upon 5-FAM/QXL^®^ substrate cleavage using the SensoLyte^®^ 520 Sortase A Activity Assay Kit (Anaspec, San Jose, CA, USA). The compounds were dissolved in dimethyl sulfoxide (DMSO) and diluted with distilled water until the concentration of DMSO was 1% and the solution’s intrinsic fluorescence was checked. Each compound was tested at 7 concentration levels in the range of 100–0.1 µM. According to kit protocol, the assay was performed in a 96-well plate, each well containing 10 µL test compound solution, 40 µL enzyme solution and 50 µL substrate solution. We used as controls the enzyme, a 1% DMSO solution, the substrate solution, and 4-hydroxymercuribenzoic acid (HMB) as the positive control. The enzymatic assay was performed for 60 min at room temperature and analyzed fluorometrically (SpectraMAX Gemini XS, San Jose, CA, USA) at Ex/Em = 490 nm/520 nm. All reported values are the means of duplicate experiments.

### 4.2. Molecular Docking

The molecular docking was carried out with DOCK program [57,58,59,60]. As a receptor we used crystal structure of *S. aureus* sortase A with PDB ID: 2MLM [12]. The geometry of ligands was calculated using YFF force field [61]. The hydrogen atoms were added with Open Babel v 2.4.0 [62]. Partial atomic charges of the ligands were added with Kirchhoff method [63]. 

Docking parameters were set as described earlier [64] with several modifications. In our experiments, as the active site atoms we selected the atoms of amino acid residues within 10 Å from the reference ligand—benzo[d]isothiazol-3-one. The spheres in the active site for semi-flexible ligand docking were set with DOCK sphgen software. Grid maps were calculated using Grid program, with grid spacing 0.3 Å. Proteins were represented by the all atom model. We used ‘multiple anchors’ parameter for virtual screening, the minimum of heavy atoms in the anchor was set to 6, and the maximum number of orientations was 1000.

Visual inspection of the complexes of compounds with sortase A was performed using Discovery Studio Visualizer 4.0 [65]. 

### 4.3. Antibacterial Assay

All bacteria were cultured in Cation-adjusted Mueller Hinton broth (CAMHB) at 37 °C overnight. A sample of each culture was then diluted 40-fold in fresh broth and incubated at 37 °C for 1.5–3 h. The resultant mid-log phase cultures were diluted (CFU/mL measured by OD_600_), then added to each well of the compound-containing plates, giving a cell density of 5 × 10^5^ CFU/mL and a total volume of 50 μL. All the plates were covered and incubated at 37 °C for 18 h without shaking.

Growth inhibition of all bacteria was determined measuring absorbance at 600 nm (OD_600_), using a Tecan M1000 Pro monochromator plate reader. The percentage of growth inhibition was calculated for each well, using the negative control (media only) and positive control (bacteria without inhibitors) on the same plate as references. The growth rates for bacteria had a variation of ±10%, which is within the reported normal distribution of bacterial growth. 

The MIC was determined as the lowest concentration at which the growth was fully inhibited, defined by an inhibition ≥80%. In addition, the maximal percentage of growth inhibition is reported as DMax, indicating any compounds with partial activity.

## Figures and Tables

**Table 1:** Structures and antibacterial activity (percentage growth inhibition) against *S. aureus* MRSA ATCC43300 for compounds **1–10** at 32 mg/L.

No.	Structure	Concentration (μM)	Antibacterial Activity on *S. aureus* MRSA ATCC43300 (%)
**1**	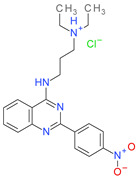	77	58.75
**2**	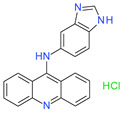	92	99.57
**3**	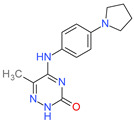	118	65.25
**4**	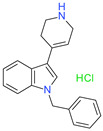	98	98.51
**5**	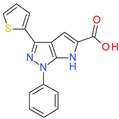	103	51.2
**6**	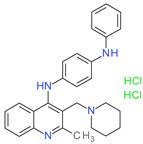	65	38.98
**7**	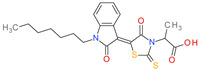	74	43.25
**8**	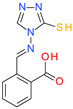	129	89.6
**9**	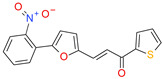	98	86.65
**10**	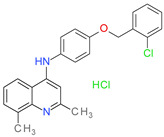	75	75.5

**Table 2:** The antimicrobial activity of compounds **1** and **2** toward multidrug resistant *S. aureus* strains.

*S. aureus* Strains	MIC (mg/L)	
	Compound 1	Compound 2
*S. aureus* 29213	78.12	156.2
MR 433	78.12	156.2
*S. aureus* 854	156.2	312.5
*S. aureus* 887	156.2	156.2
*S. aureus* 890	156.2	312.5
*S. aureus* 892	156.2	312.5
*S. aureus* 938	156.2	156.2
*S. aureus* 964	312.5	156.2
*S. aureus* 997	312.5	156.2
*S. aureus* 1012	78.12	156.2
*S. aureus* 1013	312.5	156.2
*S. aureus* 1021	156.2	156.2
*S. aureus* 584	156.2	156.2
*S. aureus* 585	156.2	156.2
*S. aureus* 586	156.2	156.2
*S. aureus* 522	156.2	156.2
*S. aureus* 523	156.2	156.2
*S. aureus* 524	156.2	156.2
*S. aureus* 501	78.12	156.2
*S. aureus* 502	78.12	156.2
*S. aureus* 503	156.2	156.2
*S. aureus* 504	78.12	156.2
*S. aureus* 505	156.2	156.2
*S. aureus* 506	78.12	156.2
*S. aureus* 507	156.2	156.2
*S. aureus* 508	156.2	156.2
*S. aureus* 509	156.2	156.2
*S. aureus* 510	78.12	156.2
*S. aureus* 511	78.12	156.2

**Table 3:** The sensitivity of *S. aureus* strains to antibiotics.

Strain	Penicillin	Oxacillin	Erythromycin	Clindamycin	Gentamicin	Chloramphenicol	Ciprofloxacin	Cefazolin	Azithromycin	Vancomycin	Linezolid	Teicoplanin	Moxifloxacin
*S. aureus* 854	R	IR	IR	S	IR	S	S	IR	IR	R	S	IR	S
*S. aureus* 887	R	IR	S	S	IR	S	S	IR	IR	IR	S	IR	S
*S. aureus* 890	R	R	R	R	R	R	IR	R	R	R	S	IR	IR
*S. aureus* 892	R	IR	S	S	IR	S	IR	S	IR	IR	S	IR	S
*S. aureus* 938	R	IR	S	S	IR	S	S	IR	IR	IR	S	IR	S
*S. aureus* 964	R	R	R	R	IR	IR	IR	S	R	IR	S	IR	IR
*S. aureus* 997	R	S	R	R	IR	IR	IR	S	IR	R	R	R	IR
*S. aureus* 1012	S	S	S	S	IR	S	S	IR	IR	R	S	IR	S
*S. aureus* 1013	R	S	IR	IR	IR	R	S	S	IR	IR	S	IR	S
*S. aureus* 1021	R	S	S	S	IR	IR	IR	IR	IR	IR	IR	IR	S
*S. aureus* 584	R	IR	R	S	IR	S	S	IR	IR	IR	S	S	S
*S. aureus* 585	R	R	IR	S	R	S	R	IR	R	IR	S	IR	IR
*S. aureus* 586	R	R	IR	S	R	R	R	R	IR	IR	S	IR	IR
*S. aureus* 522	R	R	IR	IR	R	R	R	IR	IR	IR	S	IR	IR
*S. aureus* 523	R	R	IR	IR	R	R	R	IR	IR	IR	S	IR	IR
*S. aureus* 524	R	IR	R	R	IR	S	IR	IR	R	S	S	S	S
*S. aureus* 501	R	R	R	R	IR	S	IR	S	R	S	S	IR	S
*S. aureus* 502	R	R	R	R	IR	S	IR	S	IR	IR	S	IR	S
*S. aureus* 503	R	R	R	IR	R	S	IR	S	R	R	S	IR	S
*S. aureus* 504	R	R	IR	IR	IR	S	S	S	R	IR	S	IR	S
*S. aureus* 505	R	R	R	R	IR	S	IR	S	R	IR	S	IR	S
*S. aureus* 506	R	R	R	IR	IR	S	IR	S	R	IR	S	S	S
*S. aureus* 507	R	R	R	R	IR	S	IR	S	R	S	S	S	S
*S. aureus* 508	R	R	R	IR	IR	S	IR	S	R	IR	S	IR	S
*S. aureus* 509	R	R	R	S	R	S	S	S	R	IR	S	IR	S
*S. aureus* 510	R	R	R	IR	IR	S	S	IR	R	IR	S	IR	S
*S. aureus* 511	R	IR	R	S	R	S	IR	IR	R	IR	S	IR	S

R—resistance; IR—intermediate resistance; S—susceptibility.

## Data Availability

Not applicable.

## Supplementary Materials

The following are available online, list of active compounds in SMILES format.

## Sample Availability

Samples of the compounds **1**–**10** are available from the authors.
